# Skeletal Muscle Mass Has Stronger Association With the Risk of Hyperuricemia Than Body Fat Mass in Obese Children and Adolescents

**DOI:** 10.3389/fnut.2022.792234

**Published:** 2022-03-28

**Authors:** Luyao Xie, Phoenix K. H. Mo, Qingya Tang, Xuan Zhao, Xuelin Zhao, Wei Cai, Yi Feng, Yang Niu

**Affiliations:** ^1^Department of Clinical Nutrition, Xinhua Hospital Affiliated to Shanghai Jiao Tong University School of Medicine, Shanghai, China; ^2^School of Public Health and Primary Care, The Chinese University of Hong Kong, Shatin, Hong Kong SAR, China; ^3^Shanghai Institute for Pediatric Research, Shanghai, China

**Keywords:** hyperuricemia, obesity, skeletal muscle mass (SMM), body fat mass (BFM), children

## Abstract

**Background:**

Hyperuricemia has been increasing among children with obesity in recent years. However, few studies in such a study group had explored the relationship between obesity-anthropometric indexes and hyperuricemia. This study aimed to examine the associations between hyperuricemia and different body components in children and adolescents with obesity, and further explore gender differences in these associations.

**Methods:**

In this cross-sectional study, a total of 271 obese children and adolescents (153 boys and 118 girls) aged 6–17 years were recruited from Shanghai Xinhua Hospital. Data about basic information, anthropometric assessments, body composition, and laboratory tests of participants were collected.

**Results:**

In this study, 73 boys (47.71%) and 57 girls (48.31%) were diagnosed to have hyperuricemia. The impacts of percentage of skeletal muscle (PSM) (OR = 1.221, *P* < 0.001) and skeletal muscle mass (SMM) (OR = 1.179, *P* < 0.001) on the risk of hyperuricemia was the largest, followed by hip circumference (HC) (OR = 1.109, *P* < 0.001), waist circumference (WC) (OR = 1.073, *P* < 0.001), and body fat mass (BFM) (OR = 1.056, *P* < 0.05) in whole sample, which was adjusted for age, gender and body mass index (BMI). After being stratified by gender, PSM (boys: OR = 1.309, *P* < 0.001) and SMM (boys: OR = 1.200, *P* < 0.001; girls: OR = 1.147, *P* < 0.05) were still the most predictors of hyperuricemia, followed by HC (boys: OR = 1.147, *P* < 0.001; girls: OR = 1.080, *P* < 0.05). WC showed a significant association with hyperuricemia only in boys (OR = 1.083, *P* < 0.05), while BFM showed no association with hyperuricemia in both gender groups after adjusting for age and BMI.

**Conclusion:**

Our findings suggested that SMM was a stronger predictor of hyperuricemia than BFM in children and adolescents with obesity, especially in boys.

## Introduction

The pace of the obese population has been increasing dramatically and has becoming a major public health problem. According to WHO, the prevalence of overweight and obesity increased from 4% to 18% worldwide among children and adolescents aged 5–17 years (1). The latest national estimates in China between 2015 and 2019 showed that the prevalence of overweight and obesity was 11.1% and 7.9% among 6–17-year Chinese children and adolescents, respectively (2). Obesity in childhood also raises many health concerns, such as the non-alcoholic fatty liver disease (3), type 2 diabetes mellitus (4), and cardiovascular disease (4, 5), which cause a huge burden for the health-care services and the whole society.

Serum uric acid (UA) is a weak organic acid with low solubility in water, which could be endogenously synthesized or derived from purines and their derivatives in the diet. During childhood and adolescence period, the UA level of individuals can be regulated by renal and endocrine function, diet and energy expenditure, and metabolism (6). Hyperuricemia (HUA) is caused by the imbalance of serum UA and is often accompanied by obesity and metabolic syndrome (7). In adults, children, and adolescents, HUA was found to be closely related to cardiovascular risk (8), hypertension (9), and insulin resistance (10). Therefore, it is important to further clarify the mechanism of obesity and HUA, which could help to subsequently design intervention to lower the risk of HUA and HUA-related diseases in children with obesity.

Previous studies have shown that HUA was closely associated with obesity in adults and children (10–12), but few studies focused on the associations between specific body components or anthropometric indices and the risk of HUA among the obese population. It was reported that several obesity measurements, such as body mass index (BMI), waist circumference (WC), and waist-to-height ratio (WHtR), were identified as predictors of HUA or gout in adults (13). Another obesity determiner is the U shape association between body fat percentage and prevalence of HUA among adolescent athletes (14). Despite the BMI being an overall evaluation of an individual’s body condition, it cannot differentiate the fat distribution from muscle mass, which means it cannot comprehensively assess the metabolic differences.

Some studies also suggested muscle mass and specific adiposity factors, such as WC, body fat mass (BFM), and visceral adipose tissue, were more clinically important predictors to evaluate an individual’s obesity condition and metabolic syndrome than BMI (15, 16). However, the relationships of these factors with the risk of HUA in obese children and adolescents have not been examined simultaneously to date. Therefore, identifying the associations between specific body components or anthropometric indices and HUA are particularly important for better designing interventions or treatments to ameliorate obesity and UA levels in children with obesity.

This study aimed to examine the relationship between different anthropometric indices or body components [BMI, WC, hip circumference (HC), skeletal muscle mass (SMM), and BFM] and the risk of HUA, as well as the magnitude of their effects on HUA in obese children and adolescents. Since gender differences in serum UA levels were found physiologically higher in men than in women among the children age group (17, 18), we also verified if the gender differences exist among these associations in this study. To our knowledge, this is the first epidemiologic study examining whether different body components are independently associated with HUA in children and adolescents with obesity.

## Materials and Methods

### Study Design and Participants

We conducted a retrospective study among children and adolescents, aged 6–17 years, with obesity who paid visits to the outpatient of Clinical Nutrition Department of Xinhua Hospital (Shanghai, China) from September 2012 to December 2019. The inclusion criteria for this study were the following: (a) children and adolescents aged 6–17 years old; (b) children and adolescents with obesity, wherein their BMI reached or exceeded the 95th percentile (P95) of children at the same age and gender according to the WHO standards (19). Participants with the following conditions were excluded: (a) currently receiving treatment for UA; (b) severe systemic, liver, and renal disorders; (c) taking medications that would impact obesity, such as stimulants or psychotropic drugs; (d) obesity caused by endocrine or genetic metabolic diseases. This study was approved by the Ethics Committee of Xinhua Hospital, School of Medicine, Shanghai Jiao Tong University (No. XHEC-D-2021-177).

### Data Collections

#### Anthropometric Assessments

Anthropometric measurements, including the height, body weight, WC, and HC of subjects were obtained. A vertical measuring board (Seca 264, Seca, Germany) was used to measure the height of subjects with no shoes at the floor level. Subjects were weighed on a hospital scale with indoor clothing and without shoes. WC and HC measurements were taken using a tape measure by a professional, and subjects were required to stand naturally and look straight ahead. For the WC measurement, the tape was wrapped around the waist horizontally from the midpoint between the bottom of the lower edge of the ribs and the highest point of the anterior superior iliac crest. As for measuring HC, the professional chose the most prominent part of the buttocks and then wrapped it horizontally around the buttocks using the same tape. All measurements were recorded to the nearest 0.1 cm or kg.

Body mass index, waist-to-hip ratio (WHR), and WHtR were calculated as follows: BMI was calculated as the weight in kilograms, divided by the height in meters squared (kg/m^2^); the WHR was calculated by dividing the WC (cm) by the HC (cm); the WHtR was calculated by dividing the WC (cm) by the height (cm).

The BFM and SMM of subjects were measured using bioelectrical impedance analyses (BIA) by whole-body impedance (InBody720, Biospace Inc., South Korea). The percentage of body fat (PBF) and percentage of skeletal muscle (PSM) were calculated as follows: PBF (%) = (BFM in kg)/(weight in kg); PSM (%) = (SMM in kg)/(weight in kg).

#### Laboratory Test

Blood samples were collected in the morning by experienced nurses in Xinhua Hospital with the participants in fasting state and sent to the Clinical Laboratory Center for laboratory test of serum UA. The UA values (μmol/L) were detected by the uricase method using a Hitachi 7600 automatic biochemical analyzer (Hitachi, Tokyo, Japan).

The diagnosing criteria of HUA used the reference value for serum UA in Kubota’s study (18) since UA values are highly dependent upon age. The reference cut-off levels are as follows: 5.5 mg/dL for 4–6 years, 5.9 mg/dL for 7–9 years, 6.1 mg/dL for 10–12 years in both genders, and 7 mg/dL (men) and 6.2 mg/dL (women) for 13–15 years. Furthermore, HUA was defined as above the mean UA values + 2 *SD* in each age group (16). For better comparison, we converted the UA values in this study with μmol/L to mg/dL using a conversion rate of 16.81 (1 mg/dl = 59.48 μmol/L).

#### Statistical Analysis

Data were analyzed using SPSS version 22 software (SPSS Inc., Chicago, IL, United States). Continuous variables were presented as mean ± *SD* if they were normally distributed; otherwise, median (interquartile ranges) were used. For the categorical variables, they were presented as numbers and percentages (%). To compare the difference between gender, as well as between HUA and non-HUA, independent two-tailed *t*-tests were performed for normally distributed continuous data, Wilcoxon signed-rank tests were performed for non-normally distributed continuous variables, while the Chi-square test was used for categorical variables. Binary logistic regression models were used to evaluate the impacts of different body component variables on the risk of HUA. Model 1 was an unadjusted regression; Model 2 was a regression with age and sex-adjusted; Model 3 was a regression with BMI adjusted building upon model 2. All *P*-values were calculated by two-sided tests, and the significance level for each test was set at *P* < 0.05.

## Results

### Characteristics of Subjects

A total of 271 subjects consisted of 153 boys and 118 girls were included in this study. The total mean age was 9.79 years (10.87 years in boys and 9.29 years in girls). As presented in Table 1, boys had higher height, weight, WC, HC, BMI, WHR, and WHtR in comparison with girls (*P* < 0.05). Also, boys had higher SMM and UA levels than girls (*P* < 0.05) when it comes to body components and serum UA level, while no significant differences between PSM, BFM, and HUA were found between the two groups.

**TABLE 1:** General characteristics of study subjects.

	Total (*n* = 271)	Boys (*n* = 153)	Girls (*n* = 118)	P*
Age (y)	9.79 (8.07, 11.72)	10.87 ± 2.68	9.29 ± 2.15	<0.001
Height (m)	1.44 (1.35, 1.58)	1.50 ± 0.15	1.40 ± 0.13	0.029
Weight (kg)	53.25 (43.42,71.32)	62.70 (48.60, 79.15)	46.51 (37.30, 55.74)	<0.001
BMI (kg/m^2^)	26.28 (23.51, 29.57)	27.70 (24.68, 31.11)	24.32 (21.64, 27.07)	<0.001
WC (cm)	86.00 (78.00, 96.00)	92.55 ± 12.63	80.69 ± 11.66	<0.001
HC (cm)	91.35 (83.77, 91.35)	96.93 ± 12.58	88.57 ± 11.99	<0.001
WHR	0.93 (0.89, 0.93)	0.95 ± 0.06	0.91 ± 0.07	<0.001
WHtR	0.59 ± 0.05	0.61 ± 0.05	0.57 ± 0.05	<0.001
SMM (kg)	16.80 (13.70, 22.92)	19.60 (15.35, 25.05)	14.95 (11.84, 18.49)	<0.001
PSM (%)	31.75 (30.00, 33.56)	31.84 (30.20, 33.61)	31.63 (29.68, 33.56)	0.474
BFM (kg)	25.45 (20.30, 31.77)	26.47 ± 9.58	26.70 ± 7.74	0.829
PBF (%)	39.40 (36.17, 43.20)	39.70 (37.25, 43.15)	38.70 (35.20, 43.27)	0.065
UA (mg/dl)	5.90 (5.03, 7.10)	6.30 ± 1.74	5.90 ± 1.25	0.036
HUA [*n* (%)]	130 (47.97)	73 (47.71)	57 (48.31)	0.923

*P-values were the results of the difference between boys and girls with obesity. BMI, body mass index; SMM, skeletal muscle mass; PSM, Percentage of skeletal muscle; BFM, body fat mass; PBF, Percentage of body fat; WC, waist circumference; HC, hip circumference; WHR, waist-to-hip ratio; WHtR, waist-to-height ratio; UA, uric acid; HUA, hyperuricemia.

### Comparison Between Hyperuricemia and Non-Hyperuricemia Groups

In 271 subjects, 130 of them (47.97%) were diagnosed to be HUA, among which 73 were boys while 57 were girls. As shown in Table 2, the average age in the HUA group was lower than non-HUA group, but other variables, including height, weight, BMI, SMM, PSM, BFM, WC, and HC, in the HUA group were all higher in HUA than non-HUA group (*P* < 0.05). In addition, the WHR was lower while the WHtR was higher in the HUA group than in the non-HUA group, wherein this significance only existed in boys with obesity (*P* < 0.05).

**TABLE 2:** Comparison of the body characteristics between hyperuricemia (HUA) and non-HUA groups.

	Total (*N* = 271)		Boys (*N* = 153)		Girls (*N* = 118)	
	HUA	Non-HUA	HUA	Non-HUA	HUA	Non-HUA
*N* (%)	130 (47.97)	141 (52.03)	73 (47.71)	80 (52.29)	57 (48.31)	61 (51.69)
Age (y)	9.50 (8.02, 11.35)	9.98 (8.14, 12.11)*	10.42 (8.49, 11.86)	11.29 (9.21, 13.18)^†^	9.23 (7.95, 10.33)	9.34 (7.85, 10.00)
Height (m)	1.52 ± 0.14	1.40 ± 0.13*	1.58 ± 0.14	1.43 ± 0.13^†^	1.45 ± 0.12	1.35 ± 0.13^‡^
Weight (kg)	63.80 (50.07, 81.42)	48.31 (39.88, 60.22)*	76.78 (61.95, 92.00)	56.41 (46.18, 63.88)^†^	55.72 (42.34, 65.42)	44.89 (34.77, 51.80)^‡^
BMI (kg/m^2^)	27.56 (24.05, 31.28)	25.18 (22.74, 27.70)*	30.07 (26.06, 33.02)	26.82 (24.51, 28.78)^†^	25.94 (22.52, 28.49)	23.45 (20.80, 25.86)^‡^
SMM (kg)	19.89 (15.77, 26.20)	15.12 (12.10, 19.27)*	25.69 (18.15, 33.20)	17.61 (14.18, 21.15)^†^	17.49 (13.81, 20.10)	14.29 (10.97, 16.78)^‡^
PSM (%)	31.97 (30.17)	31.65 (29.84, 33.35) *	32.41 (30.36, 34.84)	31.58 (29.73, 32.83)^†^	31.56 (29.57, 33.47)	31.75 (29.90, 33.66)
BFM (kg)	28.13 (23.37, 34.72)	22.68 (18.40, 28.07) *	30.03 (22.35, 37.35)	23.23 (17.60, 27.93)^†^	28.93 (24.55, 33.50)	24.63 (10.01, 28.45)^‡^
PBF (%)	39.35 (36.30, 43.46)	39.41 (35.77, 42.90)	39.20 (36.40, 43.10)	40.30 (37.90, 43.50)	39.73 ± 6.24	37.69 ± 5.85
WC (cm)	91.73 ± 13.83	83.38 ± 11.98*	97.61 ± 12.84	87.94 ± 10.57^†^	84.22 ± 11.25	77.41 ± 11.15^‡^
HC (cm)	98.35 ± 13.11	88.63 ± 11.01*	102.65 ± 12.87	91.71 ± 9.82^†^	92.85 ± 11.34	84.58 ± 11.27^‡^
WHR	0.93 ± 0.06	0.94 ± 0.07	0.95 ± 0.05	0.96 ± 0.08^†^	0.91 ± 0.07	0.92 ± 0.05
WHtR	0.60 ± 0.05	0.59 ± 0.05	0.62 ± 0.05	0.61 ± 0.05^†^	0.58 ± 0.06	0.57 ± 0.06

*Significance between HUA and Non-HUA groups in all subjects (P < 0.05).

*^†^Significance between HUA and Non-HUA groups in boys with obesity (P < 0.05).*

*^‡^Significance between HUA and Non-HUA groups in girls with obesity (P < 0.05).*

*BMI, body mass index; SMM, skeletal muscle mass; PSM, Percentage of skeletal muscle; BFM, body fat mass; PBF, Percentage of body fat; WC, waist circumference; HC, hip circumference, WHR, waist-to-hip ratio; WHtR, waist-to-height ratio.*

### Body Component Parameters and the Risk of Hyperuricemia

Binary logistic regression results were shown in Table 3, BMI [odds ratio (OR) = 1.198, 95% CI: 1.119–1.283], SMM, PSM, BFM, WC, and HC were positively associated with the risk of HUA after adjusting for age and gender among all participants (Model 2, all *P* < 0.001). Also, the SMM, PSM, BFM, WC, and HC were still positively associated with the risk of HUA after adjusting for age, gender and BMI in Model 3 (all *P* < 0.05), with the extent of PSM (OR = 1.221, 95% CI: 1.103, 1.352) > SMM (OR = 1.179, 95% CI: 1.099–1.264) > HC (OR = 1.109, 95% CI: 1.059 to 1.161) > WC (OR = 1.073, 95% CI: 1.026–1.121) > BFM (OR = 1.056, 95% CI: 1.004–1.11).

**TABLE 3:** Logistic regression about body component parameters and the risk of HUA.

	Model 1	Model 2	Model 3
Variables	OR (95% CI)	OR (95% CI)	OR (95% CI)
**Whole sample**			
BMI	1.140 (1.077, 1.207)***	1.198 (1.119, 1.283)***	/
SMM	1.138 (1.088, 1.190)***	1.192 (1.127, 1.261)***	1.179 (1.099, 1.264)***
PSM	1.106 (1.024, 1.194)*	1.097 (1.015, 1.185)*	1.221 (1.103, 1.352)***
BFM	1.089 (1.053, 1.126)***	1.097 (1.060, 1.136)***	1.056 (1.004, 1.110)*
WC	1.052 (1.030, 1.073)***	1.082 (1.054, 1.111)***	1.073 (1.026, 1.121)***
HC	1.069 (1.045, 1.094)***	1.095 (1.064, 1.126)***	1.109 (1.059, 1.161)***
**Boys**			
BMI	1.191 (1.094, 1.297)***	1.121 (1.107, 1.326)***	/
SMM	1.174 (1.103, 1.249)***	1.196 (1.116, 1.282)***	1.200 (1.097, 1.313)***
PSM	1.221 (1.084, 1.376)**	1.211 (1.073, 1.367)**	1.309 (1.129, 1.519)***
BFM	1.090 (1.046, 1.136)***	1.105 (1.057, 1.156)***	1.091 (0.979, 1.216)
WC	1.073 (1.040, 1.108)***	1.086 (1.050, 1.124)***	1.083 (1.017, 1.154)*
HC	1.091 (1.054, 1.130)***	1.104 (1.063, 1.148)***	1.147 (1.067, 1.233)***
**Girls**			
BMI	1.140 (1.037, 1.254)***	1.177 (1.057, 1.310)***	/
SMM	1.156 (1.060, 1.262)***	1.182 (1.073, 1.302)***	1.147 (1.018, 1.293)*
PSM	1.020 (0.934, 1.114)	1.018 (0.932, 1.113)	1.133 (0.971, 1.321)
BFM	1.086 (1.026, 1.149)**	1.088 (1.027, 1.151)**	1.055 (0.987, 1.127)
WC	1.059 (1.020, 1.099)**	1.075 (1.031, 1.122)***	1.060 (0.993, 1.132)
HC	1.069 (1.030, 1.110)***	1.081 (1.037, 1.128)***	1.080 (1.016, 1.148)*

*Model 1: Crude model.*

*Model 2: Whole sample: adjusted for age and gender; boys and girls: adjusted for age.*

*Model 3: Whole sample: adjusted for age, gender, and BMI; boys and girls: adjusted for age and BMI.*

*P < 0.05, **P < 0.01, ***P < 0.001. OR, odds ratio; CI, confidence interval; BMI, body mass index; SMM, skeletal muscle mass; PSM, Percentage of skeletal muscle; BFM, body fat mass; PBF, Percentage of body fat; WC, waist circumference; HC, hip circumference.

Among specific gender groups, the regression results showed that the BMI (OR = 1.121, 95% CI: 1.107–1.326), SMM, PSM, BFM, WC, and HC were all positively associated with the risk of HUA after adjusting for age in boys with obesity (all *P* < 0.001). On the other hand, after adjusting for both age and BMI in Model 3, the PSM (OR = 1.309, 95% CI: 1.129, 1.519), SMM (OR = 1.2, 95% CI: 1.097–1.313), HC (OR = 1.147, 95% CI: 1.067–1.233), and WC (OR = 1.083, 95% CI: 1.017–1.154) were still positively associated with the risk of HUA in boys (*P* < 0.05).

Among girls with obesity, the BMI (OR = 1.117, 95% CI: 1.057–1.31), SMM, BFM, WC, and HC were also positively associated with the risk of HUA after adjusting for age (all *P* < 0.01). Whereas after adjusting for age and BMI, only the SMM (OR = 1.147, 95% CI: 1.018–1.293) and HC (OR = 1.08, 95% CI: 1.016–1.148) were still positively associated with the risk of HUA (*P* < 0.05) in the said study group.

## Discussion

In this study, we found that the risk of HUA was positively associated with PSM, SMM, HC, WC, and BFM in obese children after controlling for age, gender, and BMI. When we analyzed the data by gender, we found that PSM, SMM, HC, and WC were still positively associated with HUA in boys, while SMM and HC were also still positively associated with HUA in girls, after adjusting for age and BMI. Specifically, the impacts of SMM (especially PSM) and HC on the risk of HUA were the largest and second in children and adolescents with obesity, in which these results were consistent after stratified by gender.

Obesity, generally defined as abnormal or excessive fat accumulation, has an adverse impact on an individual’s health (20). In our study, the proportion of obese children with HUA was 47.97% (47.71% in boys and 48.31% in girls), which was higher than some previous studies which documented around 20% HUA among obese children (7). BMI increased overall 19.8% risk of HUA in children and adolescents with obesity in this study. The relationship between obesity and HUA was interactional and complex. For example, HUA can be caused by obesity through increasing the UA synthesis and inhibiting its excretion, but it consequently results to a high level of UA which also develops obesity by accelerating body fat accumulation (especially visceral fat) (21). BMI reflects the overall body condition and is shown to be positively associated with UA levels or the risk of HUA in previous studies (22, 23). In their discussions about this finding, the author usually mentioned the effects of SMM and BFM on UA, since BMI could provide potential estimates of body fat and muscle. Thus, these findings and discussions also prompted us to further clarify the associations between HUA and some body components, such as BFM and SMM.

Our findings showed that BFM and WC were positively associated with the risk of HUA in children with obesity after adjusting for age, gender, and BMI, which was consistent with some previous studies (24, 25). The mechanisms of the association between BFM and HUA have been discussed and about the following aspects: firstly, with the accumulation of body fat, the levels of several inflammation markers, such as interleukin (IL)-6 and tumor necrosis factor α (TNF-α), have increased, which were positively related to serum UA levels in the obese population (26, 27). In addition, excess adipose tissue would be a major contributor to insulin resistance (IR) or peripheral IR (28, 29), which has been confirmed as a key risk of HUA, since IR was found to have an ability to (1) directly affect the reabsorption of UA and ultimately lead to HUA (30) or (2) indirectly promote lipolysis to increase the production of nicotinamide adenine dinucleotide phosphate (an important source of UA) (31, 32). In this study, increased WC led to a higher risk of HUA than the BFM in total participants. However, stratified analysis by gender revealed that only WC still showed positively related to HUA in boys, and neither BFM nor WC showed an association with HUA in girls with obesity. As a marker of abdominal fat accumulation and visceral adiposity in children and adolescents (33, 34), WC showed a greater association with HUA than BFM (a relative overall indicator of body fat). Such findings further elucidated visceral adipose accumulation was more closely related to HUA in children and adolescents, especially in boys, which was consistent with previous studies in adults (23–25).

For children and adolescents with simple obesity, it was less noticed that the muscle mass also increased greatly as a fat mass did (35). The first study about the association between muscle mass and UA levels in 823 Brazilian children and adolescents, which was published in 2020 and showed that a positive association between muscle mass and UA levels in both boys and girls, and muscle mass has explained the respective 43 and 7.7% of the variability of UA levels (17). In our study, it was worth noting that PSM and SMM were found to be the most powerful predictors to the risk of HUA in children with obesity, even after being stratified by gender. As known, UA is an end-product of purine catabolism from endogenous (nucleic acids and internal pool of purines, accounting for approximately 80%) and exogenous sources (dietary purines, accounting for 20%) (36) of the body. Furthermore, muscle mass is considered as the largest source of purine in the body (37). During the process of its growth, muscle cells release plenty of nucleic acids and purines due to the depletion of themselves or metabolism of adenosine triphosphate, which in turn results in increased production of UA (36, 38). Therefore, it is reasonable to attribute an increased serum UA level to an increase in muscle mass. On the other hand, increased levels of intramyocellular lipids could also elevate IR from skeletal muscles (39). Thus, we speculated that the IR may be further enhanced and the risk of HUA might increase among children with high muscle and high fat, which needs more empirical evidence to support. In our study, PSM and SMM were the most powerful predictor of the risk of HUA, followed by HC, and these magnitudes were greater in boys than in girls, which also suggested the role of muscle played in the increase of UA levels. In this study, the PSM, SMM, and HC were significantly greater in boys than girls. Larger HC usually reflects higher muscle mass (40, 41), which might be a potential explanation of the magnitude of HC on HUA higher than WC. These findings also provided evidence about the effect of muscle on HUA.

To our best knowledge, this is the first study that showed that SMM had a stronger association with the risk of HUA than BFM in obese children. Considering the gender differences in body compositions and UA levels, boys and girls were also stratified in the analysis to ensure the reliability of the results. The results among these study groups were mostly consistent to support muscle as the most powerful predictor to HUA in our study, especially in boys with greater muscle mass. However, several limitations also need to be mentioned. Firstly, due to the cross-sectional design of this study, our findings cannot be regarded as causal inferences between different body components and UA levels or HUA. Longitudinal studies are warranted to examine their relationships. Secondly, as mentioned before, part of UA can also be catabolically produced from exogenous sources (e.g., dietary purines), which means lack of dietary data about protein intake and analysis about its impact on HUA could also be a limitation. Despite this, the body composition can be regarded as an indirect reflection of dietary and nutritional status.

To be concluded, not only the BMI, higher PSM, SMM, HC, WC, and BFM were found to be positively associated with a higher risk of HUA independently in children with obesity. PSM and SMM were stronger predictors than BFM in HUA in obese children, especially in boys. The relationships between obesity and HUA are more complex than it appears. Thus, in clinical practice, more attention should be paid only not to BFM and BMI, but also to muscle mass in children with obesity (especially in boys) in order to reduce the risk of HUA and potential related metabolic diseases. Moreover, further prospective studies are warranted to clarify whether a reduction of muscle mass and fat mass could reduce UA levels, and the underlying mechanisms of the relationships among them still need more evidence.

## Data Availability Statement

The raw data supporting the conclusions of this article will be made available by the authors, without undue reservation.

## Ethics Statement

The studies involving human participants were reviewed and approved by the Ethic Committee of Xinhua Hospital, School of Medicine, Shanghai Jiao Tong University. Written informed consent from the participants’ legal guardian/next of kin was not required to participate in this study in accordance with the national legislation and the institutional requirements.

## Author Contributions

LX, YN, PM, WC, and YF conceptualized and designed the study, drafted the initial manuscript, and reviewed and revised the manuscript. XZ, XLZ, YN, and QT designed the data collection instruments, collected data, carried out the initial analyses, and reviewed and revised the manuscript. All authors contributed to the article and approved the submitted version.

## Conflict of Interest

The authors declare that the research was conducted in the absence of any commercial or financial relationships that could be construed as a potential conflict of interest.

## Publisher’s Note

All claims expressed in this article are solely those of the authors and do not necessarily represent those of their affiliated organizations, or those of the publisher, the editors and the reviewers. Any product that may be evaluated in this article, or claim that may be made by its manufacturer, is not guaranteed or endorsed by the publisher.
